# DEFIF-Net: A lightweight dual-encoding feature interaction fusion network for medical image segmentation

**DOI:** 10.1371/journal.pone.0324861

**Published:** 2025-05-29

**Authors:** Zhanlin Ji, Shengnan Hao, Quanming Zhao, Zidong Yu, Hongjiu Liu, Lei Li, Ivan Ganchev

**Affiliations:** 1 College of Mathematics and Computer Science, Zhejiang A&F University, Hangzhou, Zhejiang, China; 2 TRC/ECE, University of Limerick, Limerick, Ireland; 3 Hebei Key Laboratory of Industrial Intelligent Perception, North China University of Science and Technology, Tangshan, Hebei, China; 4 Department of Cardiology, Tangshan Workers’ Hospital, Tangshan, Hebei, China; 5 Faculty of Mathematics and Informatics, University of Plovdiv “Paisii Hilendarski”, Plovdiv, Bulgaria; 6 Institute of Mathematics and Informatics—Bulgarian Academy of Sciences, Sofia, Bulgaria; Khalifa University of Science and Technology, UNITED ARAB EMIRATES

## Abstract

Medical image segmentation plays a crucial role in computer-aided diagnosis. By segmenting pathological tissues in medical images, doctors can observe anatomical structures more clearly, thereby achieving more accurate disease diagnoses. However, existing medical image segmentation networks have issues such as insufficient capability to extract features from target areas, as well as high number of parameters and increased computational complexity. To address these issues, a lightweight Dual-Encoding Feature Interaction Fusion network (DEFIF-Net) is proposed in this paper for medical image segmentation. Firstly, in the encoding stage of DEFIF-Net, a global dependency fusion branch is introduced as an additional encoder to capture distant feature dependencies, whereby the neighboring and distant feature dependencies are effectively integrated by the newly designed feature interaction fusion convolution. Secondly, between the encoder and decoder, channel feature reconstruction modules (CFRMs) are used to enhance the feature representation of important channels. Additionally, a novel multi-branch ghost module (MBGM) is used in the bottleneck layer of the network to enhance its efficiency in capturing and retaining different types of feature information. Lastly, a novel residual feature enhancement (RFE) decoder is utilized to emphasize boundary features, thereby increasing the network’s sensitivity to lesion boundaries. The segmentation performance of the proposed DEFIF-Net network is evaluated in two different medical image segmentation tasks. The obtained experimental results demonstrate that, compared to state-of-the-art networks, DEFIF-Net exhibits superior segmentation performance on all three datasets used, while also having a lower parameter count and computational complexity.

## 1 Introduction

Medical image segmentation is a crucial step in the skin lesion segmentation process. It can segment important pathological areas from images, assisting doctors in clinical judgments [1]. Traditional skin lesion segmentation methods overly rely on doctors manually extracting features from images, which significantly increases labor and time costs [2]. Therefore, in order to enhance the efficiency and performance of skin lesion image segmentation and reduce dependence on manual intervention, automated segmentation methods have become the focus and trend of current research [3].

In recent years, researchers have been actively exploring automated skin lesion image segmentation methods based on deep learning. These methods leverage deep neural networks to automatically learn representations of features in images, providing more reliable support for diagnosis and treatment of skin diseases [4,5]. In 2015, Ronneberger et al. [6] proposed the U-Net network to tackle the semantic segmentation challenges in medical image segmentation tasks with limited sample sizes. U-Net adopts an encoder−decoder architecture and introduces skip connections between the encoder and decoder, which allows it to leverage limited sample data more effectively, leading to excellent performance on small-sample datasets such as those containing medical images. The unique design of U-Net has also inspired many subsequent networks, such as MultiResUNet [7], UNET 3+ [8], etc. They adopt similar structures to enhance their adaptability to small sample data and improve their ability to perceive subtle features in medical images. However, their methods of processing input images are somewhat limited, especially when dealing with structurally complex skin disease images, which may result in inadequate modeling of long-range dependencies [9]. To better capture long-distance dependencies between sequences, Chen et al. [10] combined the structures of Transformer and U-Net [6], designing a Transformer-based medical image segmentation network, called TransUNet, which employs a self-attention mechanism to capture global dependencies while also retaining the advantages of U-Net, enabling it to more accurately capture pathological structural information in medical image segmentation tasks. However, since Transformer-based models typically contain a large number of parameters and complex computational structures, they require more computational resources and time for training and inference, which limits their scalability and practicality in real-world applications [11]. In addition, some lightweight skin lesion segmentation models (such as I-UNeXt [12], MALUNet [13], etc.) have lower parameter count and processing speed, but perform poorly in segmentation tasks and exhibit low generalization ability. Currently, despite researchers’ continuous exploration and experimentation with new methods to improve the performance and efficiency of skin lesion image segmentation, striking a balance between segmentation performance and efficiency remains a huge challenge.

To simultaneously lower the computational costs and improve the segmentation performance, this paper proposes a novel lightweight medical image segmentation network, named DEFIF-Net. The proposed network significantly enhances the feature capture and integration capabilities through a dual encoding mechanism, and introduces a novel RFE decoder to optimize the feature space, thereby improving its accuracy in handling regions with high detail complexity in images. Additionally, by integrating specially designed lightweight modules, DEFIF-Net is able to effectively acquire complex feature information from images within low-computational environments.

The main contributions of this article are as follows:

1. A novel global dependency fusion (GDF) branch is elaborated as a second encoder in the proposed network to capture the long-range feature dependencies that could be otherwise missed. Additionally, in this branch, a novel feature interaction fusion convolution (FIFConv) is utilized to fully integrate the dependency relationships between features at different distances, thereby enhancing the network’s understanding of the overall image structure.
2. A novel channel feature reconstruction module (CFRM) is designed for the proposed network to break the independence between feature channels and promote information transmission between channels.
3. A novel RFE decoder is designed to enhance the proposed network’s perception and discrimination ability of subtle boundary features, thereby allowing it to effectively capture the boundary texture features of pathological regions.
4. A novel multi-branch ghost module (MBGM) is elaborated and utilized at the bottleneck layer of the proposed network. This module is capable of preserving feature diversity and reducing information loss while maintaining low computational complexity.

## 2 Methods

### 2.1 Related work

Medical image segmentation plays a crucial role in computer-aided diagnosis, providing essential support for lesion detection, tissue analysis, and surgical planning. In recent years, deep learning networks, particularly Convolutional Neural Networks (CNNs) [14], have made significant advancements in the field of medical image segmentation. U-Net [6] is one of the earliest and most widely used networks for medical image segmentation. It employs an encoder−decoder structure and utilizes skip connections to pass detailed features between the encoder and decoder, thus retaining more spatial information in low-resolution features. Subsequently, variants like UNet++ [15] and Attention U-Net [16] have further enhanced the feature representation capabilities of U-Net. UNet++ introduces nested and dense skip connections to capture more details, while Attention U-Net adaptively focuses on regions of interest through attention mechanisms, thus improving segmentation performance. Subsequently, in order to improve the ability to segment skin lesions, Ibtehaz et al. introduced the Inception block [17] into the U-Net network and designed an enhanced U-Net based network, called MultiResUNet [7]. Compared with other methods that directly increase the depth and width of the network, MultiResUNet can effectively utilize parameters, reduce redundancy, and improve efficiency of skin lesion segmentation, which makes it a particularly good representative of skin lesion segmentation networks. In terms of lightweight skin lesion segmentation network design, networks like MALUNet [13], EGE-UNet [18], and VM-UNet [19] offer high computational efficiency, making them suitable for applications with real-time requirements. In a recent study, Ruan et al. [20] have designed MEW-UNet by replacing the self-attention in ViT with a newly designed Multi-axis External Weights block, which achieved excellent performance on multiple medical image datasets. Chen et al. [21] designed SCSONet to balance the computational efficiency and segmentation effect of skin lesion segmentation. SCSONet effectively enhances the recognition of irregular skin lesion areas and reduces the computational burden by learning complementary attention weights. Although these networks have improved segmentation efficiency, they have not yet reached a satisfactory level in terms of segmentation performance. Currently, despite the progress made by many such networks, challenges remain in skin lesion image segmentation, particularly in handling fine structures, complex backgrounds, and cross-dataset generalization. This paper builds upon the encoder−decoder network structure to further improve feature fusion strategies and structural design, achieving a better balance between performance and inference efficiency. These innovations enhance segmentation performance and demonstrate broad applicability in complex medical imaging scenarios.

### 2.2 DEFIF-Net

#### 2.2.1 Overall structure.

The proposed DEFIF-Net network has a unique asymmetric encoder−decoder structure. In order to enhance the network’s expression of complex pathological features and optimize its ability to parse pathological structures in images, newly designed modules are integrated into DEFIF-Net to improve its segmentation performance and robustness. These innovative design enables DEFIF-Net to maintain high-performance segmentation even when operating within resource-constrained environments, significantly improving its applicability in the field of medical image segmentation. The specific implementation details of these modules are described in the following subsections.

**Fig 1:**
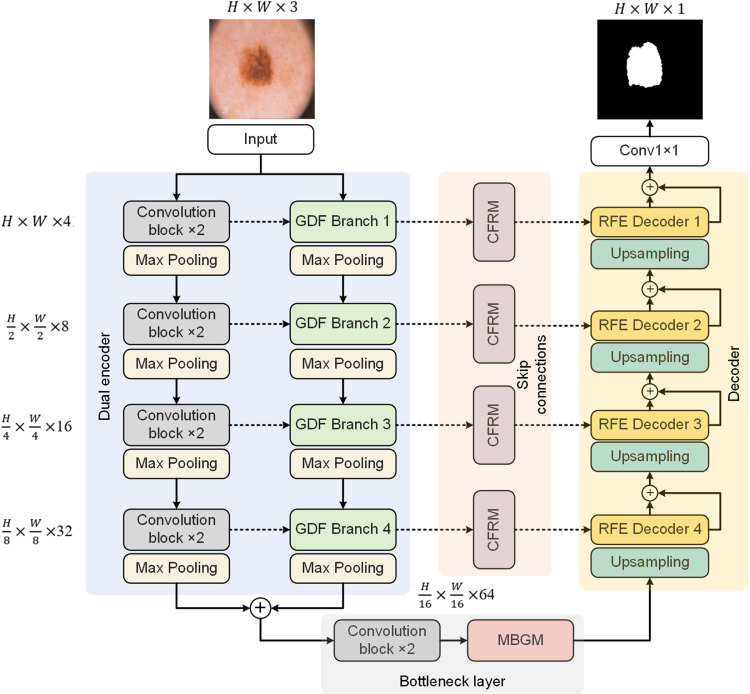
The DEFIF-Net’s overall structure.

The DEFIF-Net structure consists of four main parts: a dual encoder, a bottleneck layer, skip connections, and RFE decoders, as illustrated in Fig 1. Initially, the network receives an input feature map of size H×W×3 and feeds this into the dual encoder, where each encoding layer performs layer-wise bilinear downsampling on the input feature map through two different branches: (1) the original U-Net encoder [6] (the left part of the dual encoder), responsible for extracting local feature dependencies in the input feature map; and (2) the newly designed GDF branch (the right part of the dual encoder), focused on capturing long-range feature dependencies in the feature map. These two types of dependencies are fused after each encoding layer to form a richer feature representation. At the end of the encoding process, the final feature maps from the two encoders are merged and passed to the bottleneck layer to further integrate and enhance the feature information. Additionally, each encoding layer is equipped with a 2×2 max pooling layer to abstract the input feature map, thereby enhancing the network’s processing efficiency. In the bottleneck layer, diverse feature information in the encoder’s output feature map is preserved through two 3×3 convolutions and a newly designed MBGM. In the skip connections between the encoder and decoder, novel CFRMs are used to further emphasize the important feature channels output by the GDF branches. By integrating an upsampling layer with the RFE decoder modules, the network’s decoding unit merges and enhances features from CFRM and the bottleneck layer, enabling the network to focus more on the boundary texture of pathological targets, thereby achieving finer boundary texture depiction. Finally, a 1×1 convolution is utilized to adjust the channel numbers based on the number of categories and generate the final binary or multi-class masks.

#### 2.2.2 Dual encoder.

In the process of processing medical images, the network’s judgment of pathological regions needs to comprehensively consider both local features and spatial features [22]. Among these, the local features contain structural and textural information about the lesion areas, which are critical for identifying the type and characteristics (e.g., benign, malignant, and severity) of the lesion areas. Spatial features provide a network with overall structural information of the pathological area and its relationship with the surrounding normal skin positions, helping the network recognize pathological areas of different shapes and accurately locate lesion positions [23]. Based on this, a novel GDF branch was designed specifically to capture long-distance feature dependencies, forming complementary relationship with the output of the U-Net original encoder, thus enabling the network to simultaneously focus on both local and spatial features.

**Fig 2:**
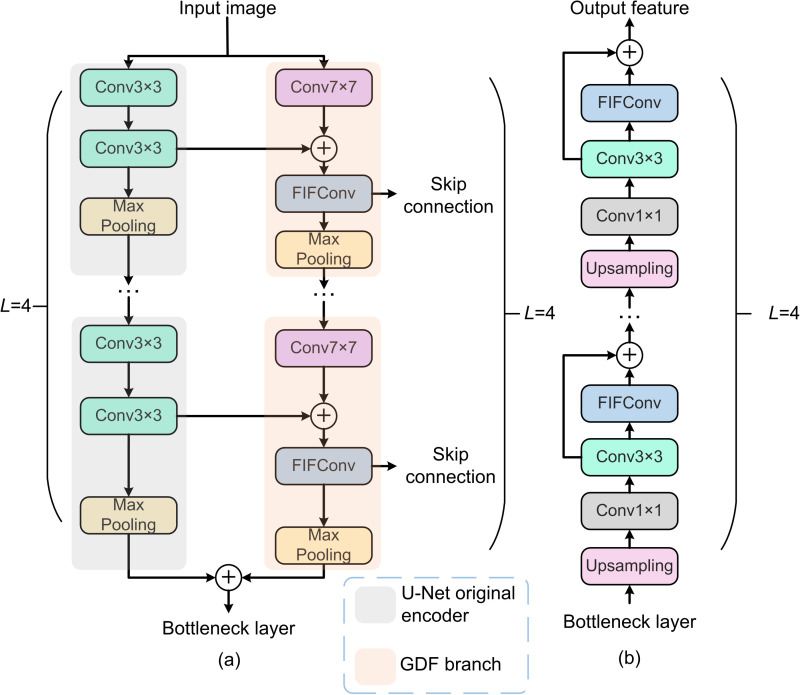
The DEFIF-Net’s: (a) dual encoder; (b) RFE decoder.

The structure of the DEFIF-Net dual encoder is shown in Fig 2a. First, the input image is separately fed into the U-Net original encoder and the newly proposed GDF branch. In the former, adjacent feature dependencies within a smaller receptive field are captured through two 3 × 3 convolution layers, and the feature maps are dimensionally reduced by a max pooling layer. Meanwhile, a 7×7 convolutional is utilized by the GDF branch to extract remote feature dependencies from a broader receptive field, which are then fused with the adjacent feature dependencies captured by the U-Net original encoder. However, simple element-wise addition fusion operations may overlook deeper feature dependencies, potentially limiting the network’s ability to comprehend global information [24]. Therefore, a novel type of convolution, FIFConv, is introduced in the GDF branch to further fuse and extract features, aiming to explore the dependencies between features. Then, the fully fused feature maps are sent separately to the max pooling layer and the skip connection section, completing a layer of feature encoding. In DEFIF-Net, this encoding process is repeated L times (in the conducted experiments, L was set to 4). After completing the feature encoding of the last layer, the final output features from both the U-Net original encoder and the GDF branch are fused and passed to the bottleneck layer of the network. This innovative design significantly enhances the network’s ability to delineate lesion boundaries, improving the segmentation performance and robustness w.r.t. complex lesion morphologies.

#### 2.2.3 RFE decoder.

As the boundary textures of pathological regions in medical images often exhibit diversity, accurately identifying the boundaries of these regions is crucial for improving the network segmentation performance [25]. Therefore, we combined FIFConv with residual structures to design a novel RFE decoder (Fig 2b), as to help the network better extract and emphasize complex boundary features, thereby improving its segmentation performance. Additionally, the residual branch also benefits the network in converging more easily during the training, thereby enhancing the network training efficiency.

Firstly, the RFE decoder restores the compressed feature maps through upsampling and adjusts the channels using 1×1 convolutions to match the size of the feature maps after the CFRM processing. Subsequently, the adjusted feature map is combined with the feature map passed through skip connections to form a richer feature representation. Next, multi-level semantic features are obtained through 3×3 convolutions, and FIFConv is utilized to enhance and refine the boundaries of target regions, aiding the network in better extracting boundary features. Finally, a residual branch is combined with feature maps after the initial fusion to emphasize important features, thereby further improving the network’s ability to express boundary features. During the decoding stage, the above process is repeated L times.

#### 2.2.4 FIFConv.

**Fig 3:**
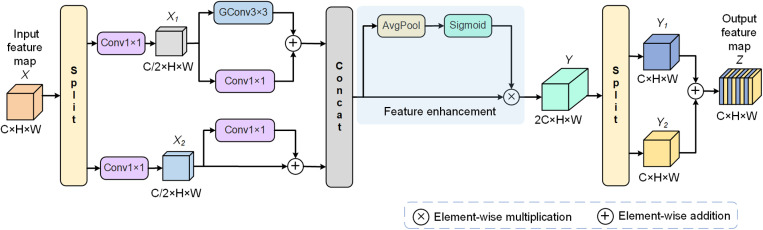
The elaborated FIFConv structure.

When integrating local and spatial features in the GDF branch, using only an element-wise addition might ignore potential relational patterns and pathological structures among the features, thus limiting the network’s analytical and understanding capabilities w.r.t. input images. Directly adding feature maps may lead to imbalance in feature importance, particularly when there are conflicts or overlaps between features, causing some features to be overemphasized or underestimated [26]. Therefore, to fully integrate detail features and spatial features, we propose here the feature interaction fusion convolution (FIFConv), shown in Fig 3, for capturing rich dependency relationships among features.

FIFConv employs a unique feature extraction strategy of tearing, fusion, tearing again, and recombining, effectively achieving interactive fusion of features from different scales and spatial extents. Specifically, for the input feature map *X* ∈ ℝ^C×H×W^, it is first equally divided into two sub-feature maps along the channel dimension during the tearing stage. Then, the sub-feature maps are transformed and fused using an 1×1 convolution to generate new feature maps $\left\{X_{1},\;X_{2}\right\}$. Subsequently, grouped convolutions are used to achieve deep learning on features within each channel group of $X_{1}$ with lower computational cost, and adding them to the feature maps processed by the 1×1 convolution. Simultaneously, feature map $X_{2}$ is enhanced through residual connections to preserve more original feature information. Subsequently, these two feature maps are fused along the channel dimension and processed through an average pooling and a sigmoid activation function. The calculated weight coefficients are then used to adjust the importance of each channel, thereby refining the expression of features. Finally, to prevent the network from overly relying on information from specific channels, the merged feature map Y is once again torn along the channel dimension and reassembled through an element-wise addition to enhance the synergistic effect among different channel features, and the output feature map *Z* ∈ ℝ^C×H×W^ from FIFConv is obtained. The above process is represented as follows:


$$\;X_{1},X_{2}=S\left(X\right)$$ (1)

$$X_{1},X_{2}=C_{1}\left(X_{1}\right),C_{1}\left(X_{2}\right)$$ (2)

$$X_{1}=G_{3}^{2}\left(X_{1}\right)+C_{1}(X_{1};$$ (3)

$$X_{2}=X_{2}+C_{1}(X_{2};$$ (4)

$$Y_{1},Y_{2}=S(X_{1}\;||\;X_{2}\times \sigma (A(X_{1}\;||\;X_{2}));$$ (5)

$$Z=Y_{1}+Y_{2}\;$$ (6)

where S and || denote splitting and merging feature maps along the channel dimension, respectively, × denotes an element-wise multiplication, $C_{k}$ denotes a regular convolution with a kernel size k, $G_{k}^{n}$ denotes a grouped convolution operation with a k×k kernel size dividing the input channels into n groups, σ denotes the sigmoid activation function, and A denotes an average pooling.

#### 2.2.5 CFRM.

**Fig 4:**
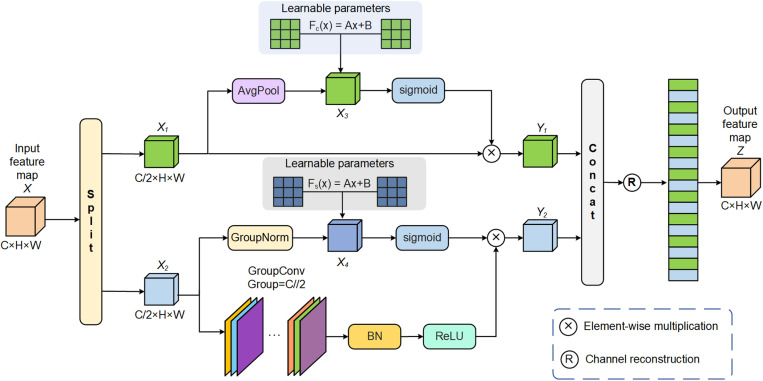
The elaborated CFRM structure.

By selecting task-relevant channels from the feature map and enhancing their performance, the interference of irrelevant information on the decoder can be reduced [27]. Therefore, to break the isolation between channels in the feature map, promote interaction between channels, and enhance the network’s utilization of important channel features, we designed a novel CFRM module with the structure depicted in Fig 4. To avoid increasing the number of network parameters, one branch in CFRM uses an average pooling to embed global features, while the other branch employs group normalization to enhance the network’s understanding of spatial relationships and pathological structures. Subsequently, the network’s adaptability to different data is enhanced by adaptively learning the features of two different channel groups using two learnable parameters. Moreover, by enhancing and reconstructing features of each channel, CFRM allows the proposed network to concentrate more on learning and extracting critical channel features, thus enhancing its sensitivity to pathological regions. Algorithm 1 details the operational process of the CFRM in a pseudocode form.

Specifically, in CFRM, to reduce the computational burden on the network, the feature map X is first divided into two sub-channel groups $\left\{X_{1},\;X_{2}\right\}$ along the channel dimension, and different feature extraction operations are performed on these two sub-channel groups. In the first feature extraction branch, to avoid increasing the network’s parameter count, an average pooling is used to embed global features. Then, by using a learnable parameters to adaptively adjust the network’s selection of different features, the network can more flexibly capture subtle differences in various data, thereby maintaining high segmentation performance. Next, the importance of each pixel is calculated using the sigmoid function to enhance the network’s focus on important features, while reducing its attention to background information (irrelevant features) in the images.

In the other feature extraction branch, a 3×3 grouped convolution is used to enhance the feature representation capabilities within each channel group. Additionally, group normalization is used to improve the network’s understanding of spatial relationships and structures, and learnable parameters along with the sigmoid function are used to dynamically adjust feature weights. Following this, by multiplying the two feature maps in the second branch, the network’s attention to important channels is enhanced, resulting in feature map $Y_{2}$. Then, feature maps $Y_{1}$ and $Y_{2}$ are concatenated, and the channel order of the feature maps is randomly shuffled to promote information exchange between feature maps, resulting in the output feature map Z of CFRM.

Algorithm 1: The PyTorch-style pseudo-code of CFRM

**#**Input: **X**, the feature map with shape [B, C, H, W]

**#**Output**: Z**, the feature map with shape [B, C, H, W]

**#**Operator: **AP**, Average pooling operation

**     GN**, Group normalized

**     Sigmoid**, Sigmoid function

1: X1, X2 = X. chunk (2, dim=1)

2: Y1 = X1 * **Sigmoid** (**AP** (X1) * Parameter (torch.zeros (1, C// 2, 1, 1)) + Parameter (torch.ones (1, C// 2, 1, 1)))

3: Y2 = Conv1 (X2) * Sigmoid (**GN** (X2) * Parameter (torch.zeros (1, C// 2, 1, 1)) + Parameter (torch.ones (1, C// 2, 1, 1)))

4: Y = torch.cat ([Y1, Y2], dim=1)

5: B, C, H, W = Y. shape

6: Z = Y. reshape (B, 2, -1, H, W). permute (0, 2, 1, 3, 4). reshape (B, -1, H, W)

#### 2.2.6 MBGM.

**Fig 5:**
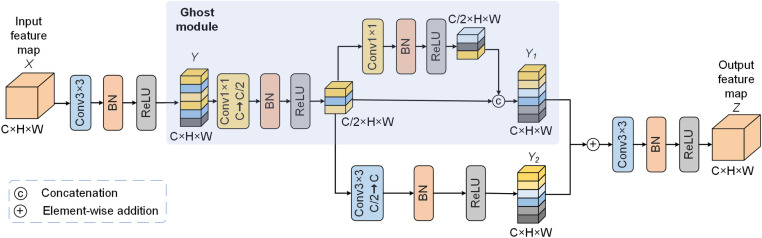
The elaborated MBGM structure.

Convolution operations have issues with fixed receptive fields and insufficient information fusion [28]. Therefore, employing regular convolutions in the bottleneck layer fails to adequately capture the feature representations in the encoder [29]. To address this issue, a newly designed MBGM is introduced in the bottleneck layer of the proposed network. The MBGM design was inspired by the Ghost module [30], which utilizes a parallel combination of 1×1 convolutions and 3×3 convolutions, allowing each branch to focus on capturing different information from the input features. This enables the network to better capture and preserve diverse feature information with lower computational cost. The elaborated MBGM structure is shown in Fig 5.

MBGM first extracts higher-level feature information from input feature map X by using a 3×3 convolution to generate feature map Y. Afterwards, feature map Y is passed to the Ghost module, where two sets of 1×1 convolutions and residual branches are used to merge the feature information of the two feature maps. To further extract local features, an additional feature extraction branch is used after the first convolution in the Ghost module, and the extracted features are fused with feature map $Y_{2}$. Finally, the feature map is refined through a 3×3 convolution. Additionally, in MBGM, batch normalization and ReLU activation functions are utilized after each convolution to introduce non-linearity characteristics and maintain network stability. MBGM effectively integrates feature information from the encoder and preserves relatively complete feature representations. This innovative design improves the segmentation performance of the proposed network w.r.t. various pathological images, while maintaining efficient network operation. The MBGM processing is represented as follows:


$$Y=C_{3}\left(X\right)$$ (7)

$$Z=C_{3}[C_{1}\left(C_{1}\left(Y\right)\right)||\;C_{1}(Y;+\;C_{3}\left(C_{1}\left(Y\right)\right)]$$ (8)

## 3 Experiments and results

### 3.1 Datasets and data preprocessing

Three datasets were used in the experiments conducted to evaluate the segmentation performance of DEFIF-Net in comparison to state-of-the-art networks. The two datasets used in the first group of experiments were the ISIC2016 [31] and ISIC2017 [32] public skin disease datasets, provided by the International Skin Imaging Collaboration, containing 1279 and 2150 images, respectively. These are two important resources in the field of skin disease research, with a certain level of authority. Additionally, we evaluated the generalization performance of DEFIF-Net, in comparison to state-of-the-art networks, in various segmentation tasks using the DSB2018 public microscopic cell dataset.

In the experiments, the aforementioned datasets were randomly split into training, validation, and test sets at a ratio of 7:2:1, as specified in Table 1. Additionally, in order to address overfitting in the training process and the dataset imbalances, we employed data augmentation techniques such as random rotation, flipping, cropping, and adding random body-hair noise during image preprocessing. These operations can expand the representation of the lesion area and increase the network robustness to foreground lesions.

**Table 1:** Splitting the utilized datasets into training, validation, and test sets.

Dataset	Training set	Validation set	Test set	Total
ISIC2017	1548	387	215	2150
ISIC2016	921	230	128	1279
DSB2018	483	120	67	670

### 3.2 Experimental environment

DEFIF-Net was developed on a computer equipped with a Xeon(R) Platinum 8255C CPU and an RTX 3080 GPU, using PyTorch version 3.9. In the experiments, to ensure that the network is adequately trained, we adopted the following settings: the number of epochs was set to 200 (as the network training effect remained stable after reaching this number), the batch size was set to 8, the initial learning rate was set to 0.0001, the learning rate decay factor was one-half, and an improved combined binary cross entropy (BCE) [33] − Dice [34] loss function was used for network training, represented as:


$$\mathrm{\backslash [}L=0.7*\left(1-\frac{2\sum {\mathrm{\backslash nolimits}}_{i=1}^{N}p_{i}g_{i}}{\sum {\mathrm{\backslash nolimits}}_{i=1}^{N}p_{i}^{2}+\sum {\mathrm{\backslash nolimits}}_{i=1}^{N}g_{i}^{2}}\right)+0.3*\left(-\frac{1}{N}\sum_{i=1}^{N}\left(g_{i}\log p_{i}+\left(1-g_{i}\right)\log\left(1-g_{i}\right)\right)\right)\mathrm{\backslash ]}$$ (9)

where $p_{i}$ denotes the predicted value, $g_{i}$ denotes the ground-truth value, and N denotes the total number of pixels.

### 3.3 Evaluation metrics

In the experiments conducted, the Intersection over Union (IoU), Dice Similarity Coefficient (DSC), accuracy (Acc), and recall (Rec) were used to objectively evaluate the segmentation performance of the proposed network in comparison to state-of-the-art networks.

### 3.4 Experimental results and analysis

#### 3.4.1 Performance comparison on ISIC2016 and ISIC2017 datasets.

In the first set of experiments, we conducted a quantitative comparison of DEFIF-Net with multiple medical image segmentation networks, performed on the ISIC2016 and ISIC2017 datasets. The networks compared include both classic networks (U-Net [6], UNet++ [15], Attention U-Net [16]) and mainstream networks (TransUNet [10], AttaNet [35], I-UNeXt [12], MALUNet [13], EGE-UNet [18], DCSAU-Net [36], MultiResUNet [7], SCSONet [21]). The experimental results obtained are shown in Table 2.

**Table 2:** Comparative experimental results, obtained on ISIC2016 and ISIC2017 datasets.

Network	ISIC2016				ISIC2017			
	IoU (%)	DSC (%)	Acc (%)	Rec (%)	IoU (%)	DSC (%)	Acc (%)	Rec (%)
U-Net	80.46 ± 0.16	88.03 ± 0.14	93.27 ± 0.11	91.57 ± 0.15	79.62 ± 0.17	87.39 ± 0.13	93.96 ± 0.07	89.19 ± 0.14
UNet++	82.31 ± 0.12	89.70 ± 0.09	94.18 ± 0.07	88.91 ± 0.10	81.44 ± 0.17	88.49 ± 0.14	94.23 ± 0.07	90.15 ± 0.15
Attention U-Net	83.25 ± 0.11	90.41 ± 0.08	94.31 ± 0.07	90.62 ± 0.11	82.33 ± 0.15	89.32 ± 0.12	94.66 ± 0.06	90.45 ± 0.13
TransUNet	82.89 ± 0.11	90.13 ± 0.07	93.98 ± 0.11	90.91 ± 0.13	82.25 ± 0.16	89.14 ± 0.13	94.85 ± 0.07	90.04 ± 0.13
AttaNet	84.24 ± 0.11	91.08 ± 0.08	95.09 ± 0.05	**94.12** **±0.08**	82.31 ± 0.16	89.20 ± 0.13	94.52 ± 0.06	90.26 ± 0.14
I-UNeXt	82.05 ± 0.11	90.28 ± 0.08	94.57 ± 0.06	93.48 ± 0.10	81.46 ± 0.13	88.58 ± 0.13	94.37 ± 0.06	**91.83** **±0.12**
MALUNet	81.49 ± 0.14	88.93 ± 0.11	93.90 ± 0.08	91.51 ± 0.12	81.32 ± 0.16	88.59 ± 0.13	94.29 ± 0.06	89.89 ± 0.15
EGEU-Net	82.92 ± 0.11	90.19 ± 0.08	94.25 ± 0.07	91.46 ± 0.11	81.48 ± 0.16	88.75 ± 0.12	94.59 ± 0.06	90.93 ± 0.14
DCSAU-Net	84.55 ± 0.10	91.26 ± 0.09	95.02 ± 0.09	90.60 ± 0.10	82.34 ± 0.15	89.02 ± 0.13	94.19 ± 0.06	90.23 ± 0.14
MultiResUNet	83.36 ± 0.10	90.58 ± 0.06	94.37 ± 0.07	91.26 ± 0.11	80.48 ± 0.18	87.70 ± 0.15	94.10 ± 0.08	88.40 ± 0.18
SCSONet	82.80 ± 0.11	90.17 ± 0.10	94.20 ± 0.07	91.48 ± 0.12	81.50 ± 0.17	88.78 ± 0.14	94.70 ± 0.06	90.11 ± 0.15
DEFIF-Net (*proposed*)	**85.06 ± 0.09**	**91.60 ± 0.06**	**95.21 ± 0.04**	91.76 ± 0.09	**83.39 ± 0.14**	**90.02 ± 0.11**	**95.17** **± 0.04**	90.80 ± 0.14

From the experimental results in Table 2, it is evident that DEFIF-Net achieved the best segmentation performance on both datasets, according to three out of four evaluation metrics including the two major ones used in the field of image segmentation, namely IoU and DSC.

Specifically, on the ISIC2016 dataset, DEFIF-Net achieved an IoU value of 85.06%, a DSC value of 91.60%, and an accuracy value of 95.21%, which are respectively higher by 0.51, 0.34, and 0.12 percentage points than those of the second best performing network (i.e., DCSAU-Net according to IoU and DSC, and AttaNet according to accuracy). Additionally, when compared to the lightweight EGEU-Net network, DEFIF-Net’s IoU, DSC, and accuracy values are better by 2.14, 1.41, and 0.96 percentage points, respectively. However, in terms of recall, DEFIF-Net slightly lags behind the leader (AttaNet) and first runner-up (I-UNeXt), thus taking third place.

On the ISIC2017 dataset, the proposed network also leads in IoU, DSC, and accuracy, achieving 83.39%, 90.02%, and 95.17%, respectively, which makes it better that the second best performing network by: 1.05 percentage points (w.r.t. DCSAU-Net) based on IoU, 0.70 percentage points (w.r.t. Attention U-Net) according to DSC, and 0.32 percentage points (w.r.t. TransUNet) based on accuracy. According to recall, DEFIF-Net takes third place, by closely following the leader (I-UNeXt) and first runner-up (EGEU-Net).

Overall, DEFIF-Net demonstrates strong competitiveness in skin lesion segmentation tasks, and its excellent performance on the ISIC2016 and ISIC2017 datasets clearly demonstrates its potential in the field of skin lesion image analysis.

**Fig 6:**
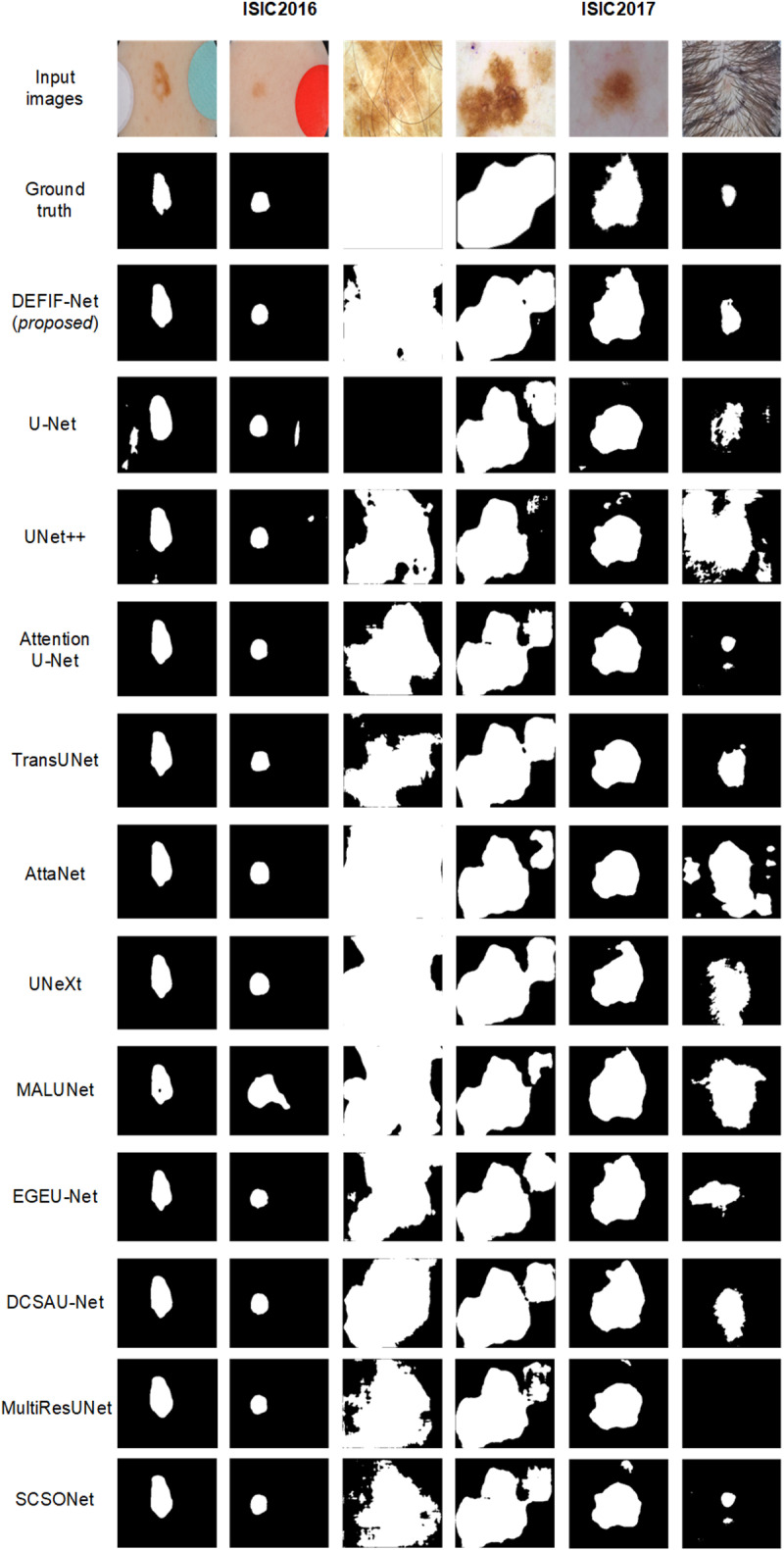
Visualization comparison of network segmentation results on the ISIC2016 and ISIC2017 datasets.

A sample visualization of the segmentation results, achieved by the compared networks on the ISIC2016 and ISIC2017 datasets, is shown in Fig 6.

Firstly, when observing the comparative images shown in the first and second columns of Fig 6, it can be noticed that DEFIF-Net effectively suppresses the background noise around the skin lesion area compared to some other networks not performing well. Secondly, from the third and fourth columns, it can be noticed that when faced with large areas of lesion regions, DEFIF-Net can more completely segment the skin lesion area, while other networks can only segment the regions with more obvious lesion features in the images. In particular, U-Net incorrectly identifies the entire image in the third column as a normal skin area. Furthermore, by observing the comparative images in the fifth column of Fig 6, it can be clearly seen that DEFIF-Net performs better than other networks in extracting boundary pixels of skin lesions. Finally, from the sixth column, displaying skin lesion images with body-hair noise, it can be seen that other networks might incorrectly identify areas with abundant body hair as lesion areas, whereas the proposed network localizes the target around the lesion area and its surroundings, resulting in the minimal occurrence of false positives. Overall, DEFIF-Net demonstrates significant superiority over other networks in all aspects, particularly in effectively suppressing background noise, segmenting large lesion areas, and extracting boundary pixels, which establishes it as a more precise tool for skin lesion segmentation.

Table 3 shows the average Hausdorff distances (HD) of different networks compared on the ISIC2016 and ISIC2017 datasets. On both datasets, the proposed DEFIF-Net network achieved the smallest average HD values of 18.60 and 19.56, respectively. Moreover, even though the average HD values of AttaNet (i.e., 18.90 on ISIC2016) and TransUNet (i.e., 19.95 on ISIC2017) are close to those of DEFIF-Net, their values fluctuate greatly. In contrast, DEFIF-Net’s HD values maintain higher stability on both datasets.

**Table 3:** The average Hausdorff distances (in pixels) of networks compared on the ISIC2016 and ISIC2017 datasets.

Network	ISIC2016	ISIC2017
U-Net	30.05	28.33
UNet++	28.27	26.86
Attention U-Net	25.59	20.87
TransUNet	23.18	19.95
AttaNet	18.90	22.10
I-UNeXt	20.52	23.63
MALUNet	23.50	20.50
EGEU-Net	25.01	24.29
DCSAU-Net	20.92	21.04
MultiResUNet	22.74	22.61
SCSONet	24.65	23.30
DEFIF-Net (*proposed*)	**18.60**	**19.56**

#### 3.4.2 Performance comparison on DSB2018 dataset.

In medical imaging, skin lesions typically manifest as singular and relatively large feature areas, whereas cell nuclei are represented as small and densely packed pathological structures. Therefore, in the second group of experiments, we selected a cell microscopy image dataset with significantly different characteristics from skin lesions to verify the generalizability of the proposed DEFIF-Net network in other medical imaging tasks. From the previous group of experiments, we selected several networks that performed well in cell segmentation tasks, including U-Net, AttaNet, I-UNeXt, MALUNet, EGEU-Net, TransUNet, and DCSAU-Net, for a quantitative comparison with the proposed network. Experimental results are presented in Table 4.

**Table 4:** Comparative experimental results, obtained on the DSB2018 dataset.

Network	IoU (%)	DSC (%)	Acc (%)	Rec (%)
U-Net	79.13 ± 0.20	86.19 ± 0.20	96.34 ± 0.04	90.22 ± 0.20
AttaNet	73.93 ± 0.19	82.97 ± 0.19	95.54 ± 0.04	84.55 ± 0.19
I-UNeXt	78.56 ± 0.20	85.84 ± 0.20	96.27 ± 0.04	85.49 ± 0.20
MALUNet	78.61 ± 0.20	85.84 ± 0.20	96.22 ± 0.04	88.36 ± 0.20
EGEU-Net	79.09 ± 0.19	86.16 ± 0.19	96.31 ± 0.03	88.03 ± 0.19
TransUNet	80.36 ± 0.18	86.90 ± 0.15	**96.53 ± 0.04**	88.08 ± 0.17
DCSAU-Net	81.07 ± 0.15	88.36 ± 0.13	96.47 ± 0.04	89.91 ± 0.14
DEFIF-Net (*proposed*)	**81.37 ± 0.12**	**89.17 ± 0.08**	96.28 ± 0.04	**91.86 ± 0.07**

As can be seen from Table 4, DEFIF-Net outperformed all other networks in the task of cell microscopy image segmentation, according to three out of four evaluation metrics, including the two major ones (IoU and DSC). Specifically, DEFIF-Net achieved an IoU value of 81.37%, a DSC value of 89.17%, and a recall value of 91.86%, which are respectively higher by 0.30, 0.81, and 1.64 percentage points than those of the second best performing network (i.e., DCSAU-Net according to IoU and DSC, and U-Net according to recall). This indicates that DEFIF-Net possesses outstanding generalization ability and versatility when dealing with different types of medical image tasks. Only according to accuracy, the proposed network was not on the top of some networks.

**Fig 7:**
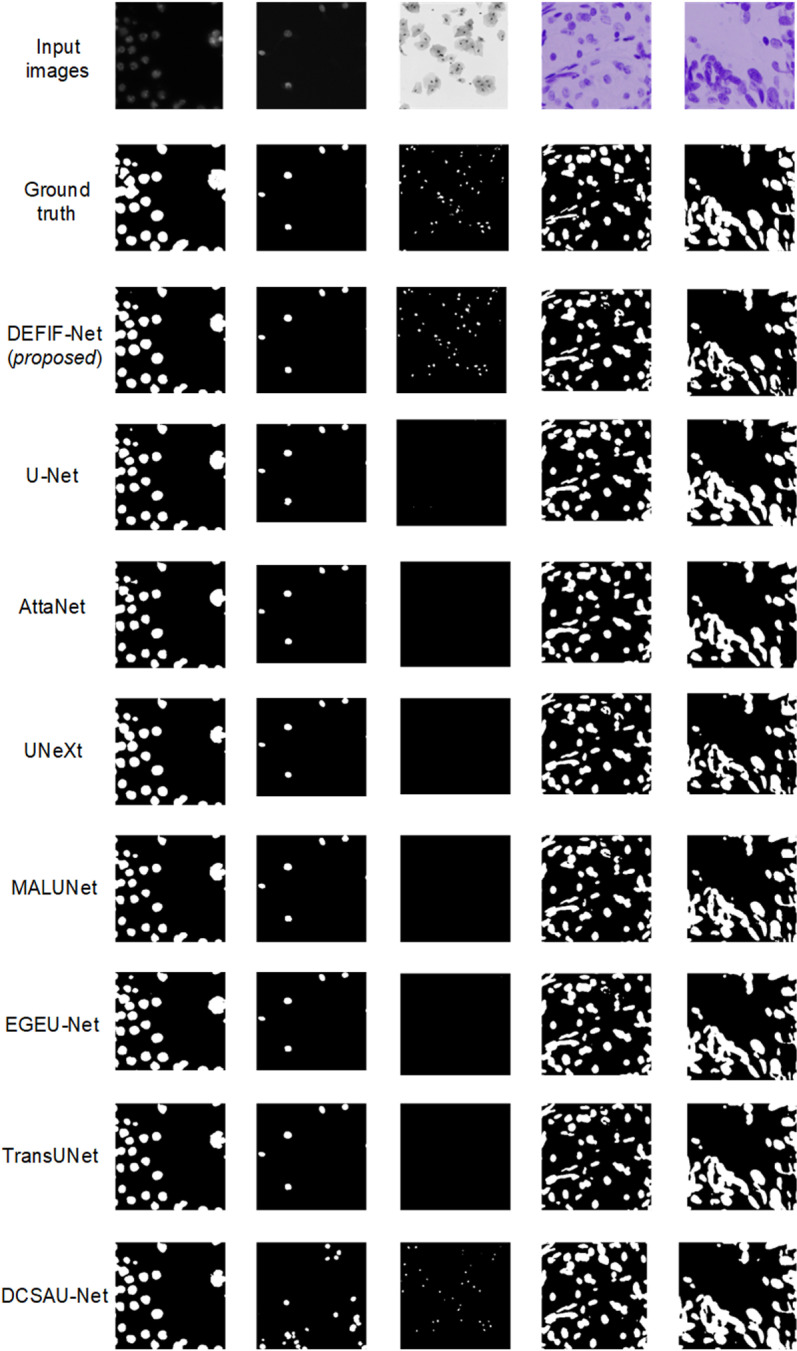
Visualization comparison of network segmentation results on DSB2018 dataset.

A sample visualization of the segmentation results, achieved by the compared networks on the DSB2018 dataset, is shown in Fig 7. This visualization further confirms the DEFIF-Net’s excellent performance in cell microscopy image segmentation tasks. Relative to other networks, DEFIF-Net achieves a more comprehensive segmentation of cells within microscopy images. Specifically, among all compared networks, only DEFIF-Net and DCSAU-Net successfully segmented the cells in the micrographs of the third column in Fig 7, but compared to DCSAU-Net, the segmentation results of DEFIF-Net are clearer. This comparison highlights the advantages of DEFIF-Net in processing fine structures, which enables it to achieve more detailed segmentation when processing microstructures.

### 3.5 Ablation study

We adopt U-Net as a baseline network for conducting extensive ablation experiments on the same three public datasets used in the other experiments, so as to explore the influence of the newly designed components on the proposed network’s performance. Table 5 presents the experimental results of the ablation study.

**Table 5:** Results of ablation study experiments.

Step	Network	ISIC2016		ISIC2017		DSB2018	
		IoU (%)	DSC (%)	IoU (%)	DSC (%)	IoU (%)	DSC (%)
0	Baseline	80.46	88.03	79.62	87.39	79.13	86.19
1	+GDFB (with FIFConv)	82.40	89.79	81.23	88.60	79.37	87.23
2	+GDFB (with standard convolution)	81.75	89.31	80.76	88.22	79.29	87.25
3	+CFRM	82.34	89.86	81.52	88.79	79.88	88.20
4	+MBGM	82.51	89.90	81.64	88.89	79.68	87.91
5	+GDFB+RFED	83.26	90.45	82.01	88.92	80.30	88.43
6	+CFRM+MBGM	83.49	90.63	82.04	89.28	80.21	88.32
7	+GDFB+RFED+CFRM	84.20	91.01	82.95	89.75	80.80	88.65
8	+GDFB+RFED+MBGB	84.65	91.38	82.90	89.58	80.88	88.93
9	+RFED+CFRM+MBGB+GDFB (with standard convolution)	83.97	90.94	82.46	89.49	80.87	88.66
10	+RFED+CFRM+MBGB+GDFB (with FIFConv), i.e., the *proposed* DEFIF-Net.	**85.06**	**91.60**	**83.39**	**90.02**	**81.37**	**89.17**

Firstly, we separately added the GDF branch (GDFB), CFRM, and MBGM to the baseline, which resulted in improvement on both metrics used in this experiment (i.e., IoU and DSC) on all three datasets, whereby the MBGM addition performed the best on ISIC2016 and ISIC2017, whereas the CFRM addition was the best on DSB2018. This indicates that these three newly designed components can effectively enhance the baseline network’s performance in segmentation tasks. Next, we retained GDFB and replaced the U-Net original decoder with the newly designed RFE decoder (RFED), which led to an additional improvement of both IoU and DSC on all three datasets. Subsequently, we incorporated CFRM together with MBGM into the baseline, which proved working better than the previous step on ISIC2016 and ISIC2017, but worse on DSB2018. In addition, to explicitly ablate the use of FIFConv by GDFB, we replaced FIFConv with a standard convolution. However, as expected, this replacement resulted in decreasing both metrics on all three datasets, except for DSC on DSB2018. This is because FIFConv fully integrates the details and spatial features, and reduces the feature loss so that there are more available features in the next stage of the network. Therefore, we refrained from applying this replacement in the following steps of the ablation study where components were added in triples to the baseline. Next, on the basis of the network structure with the dual encoder and the RFE decoder, we separately added CFRM and MBGB, and then both of them in combination (resulted in the proposed DEFIF-Net network). Experimental results show that these two components have a good synergistic effect on enhancing the network structure, thus further improving the segmentation performance. Finally, the proposed DEFIF-Net network, formed by incorporation of all four newly designed components into the baseline, achieved the highest results for both metrics on all three datasets, clearly demonstrating the effectiveness of using these novel components in the field of medical image segmentation. Just to be on the safe side, we conducted one additional experiment with all components added to the baseline, however, with GDFB using a standard convolution instead of the newly proposed FIFConv. Again, as expected, this replacement resulted in decreasing both metrics on all three datasets, which completely proved the benefit of using FIFConv instead of a standard convolution.

**Fig 8:**
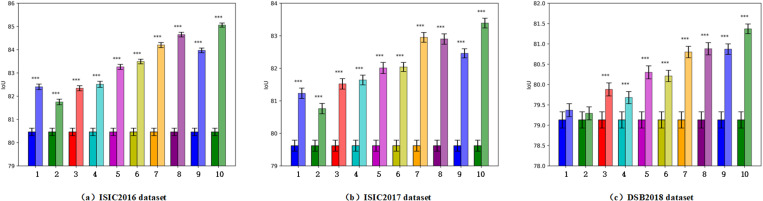
IoU significance comparison of the effect of adding the newly designed components to the baseline (the group numbers on X axis correspond to the step numbers in Table 5; the left bar of each group represents the IoU of the baseline, whereas the right bar represents the IoU of the resultant network after adding the corresponding component(s) to the baseline; the asterisks on each group represents the significance level).

Fig 8 shows the IoU significance comparison of the effect of adding the newly designed components to the baseline network (U-Net). The significance level is marked with “***” in the figure to indicate whether the difference in the network performance reached a statistically significant level. As can be seen from the figure, the addition of all components to the baseline achieved a “***” result (except for the first two steps performed on the DSB2018 dataset), indicating that each improvement in the network performance after adding the corresponding component/s is statistically significant. In addition, as components were gradually added to the network, the standard deviation gradually decreased, which further proves the important role of the newly designed components in improving the network stability.

## 4 Discussion

In the field of medical image segmentation, the networks’ parameter count and computational complexity have attracted much attention from researchers [37]. Numerous studies have been dedicated to developing deep learning networks with fewer parameters and reduced computational complexity, aimed at improving the efficiency of performing medical image segmentation tasks [38]. However, although many of these have made good improvements w.r.t. parameter count and computational complexity, their performance in image segmentation tasks is not ideal. Accurate recognition of subtle pathological structures and target boundaries typically requires networks to have strong feature representation capabilities. Networks with low number of parameters may not adequately capture this critical information, which affects their segmentation performance [39]. During the implementation of DEFIF-Net, we designed several lightweight modules aimed at enhancing the network’s ability to perceive complex structures and subtle features, as to compensate for any information loss that might occur due to the simplified network structure. The careful design of these modules allows the proposed network to achieve better segmentation performance while maintaining low parameter count and computational complexity. The experimental results obtained in this regard are shown in Table 6.

**Table 6:** Network comparison w.r.t. parameter count, floating-point operations per second (FLOPs), and inference time.

Network	Parameter count (M)	FLOPs (G)	Inference time (sec)
U-Net	7.23	12.14	10.91
UNet++	9.16	34.90	18.79
Attention U-Net	34.87	66.63	21.02
TransUNet	100.99	34.54	22.97
AttaNet	1.00	0.72	6.89
I-UNeXt	1.47	0.57	7.01
MALUNet	0.17	0.08	6.64
EGEU-Net	0.04	0.07	6.35
DCSAU-Net	2.59	6.91	8.61
MultiResUNet	18.75	7.25	19.71
SCSONet	0.14	0.05	6.73
DEFIF-Net (*proposed*)	0.24	0.33	6.75

Among the compared networks, DEFIF-Net stands out for its low parameter count of only 0.24M parameters (while also achieving a good FLOPs value of 0.33G), surpassing in this regard the lightweight networks I-UNeXt and AttaNet, which highlights the lightweight design of DEFIF-Net. In addition, Table 6 also compares the inference time of DEFIF-Net with the inference time of other networks (calculated w.r.t. all images in the test set under the same environment), compared on the ISIC2017 dataset. As can be seen from the table, w.r.t. inference time, DEFIF-Net is far ahead of mainstream networks (UNet++, Attention U-Net, TransUNet, MultiResUNet) and is comparable to the lightweight networks (AttaNet, I-UNeXt, MALUNet, EGEU-Net). This advantage not only enables DEFIF-Net to be applied in resource-constrained environments, but also effectively improves the actual deployment value of the proposed network.

**Fig 9:**
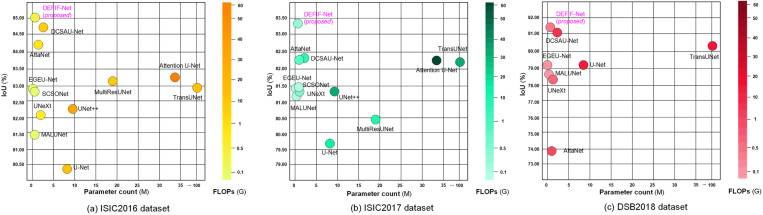
Parameter count, FLOPs, and IoU of networks, compared on: (a) ISIC2016 dataset; (b) ISIC2017 dataset; (c) DSB2018 dataset.

Through the visualization of parameter count, FLOPs, and IoU, shown in Fig 9, one can more intuitively observe the performance of the compared networks across the three datasets used. For instance, the large network TransUNet demonstrated top segmentation performance, which corresponds to its larger parameter count and FLOPs. However, it is important to note that lightweight networks show considerable variability in performance across various datasets, with good results in some of these and poorer outcomes in others, suggesting relatively weaker generalization abilities. In comparison, the proposed network exhibited the best performance on all three datasets. It not only surpassed larger networks in segmentation performance but also matched lightweight networks in terms of parameter count and FLOPs. This indicates that DEFIF-Net has achieved a good balance between segmentation performance and inference speed, offering remarkably high practicality and applicability in medical image segmentation tasks.

However, although DEFIF-Net already has fewer parameters and lower computational complexity, there is still room for further optimization. Future research will focus on exploring ways to further reduce the parameter count and computational complexity of DEFIF-Net while maintaining top segmentation performance, aiming to enhance its lightweight nature and inference efficiency.

In order to verify the impact of the loss function on the network segmentation performance, we conducted experiments with the Dice loss and BCE loss, separately and in combination using different weights for the Dice loss (α) and BCE loss (β). Table 7 shows that the best segmentation results were achieved with α=0.7 and β=0.3. However, when the weight of the Dice loss was further increased, the network segmentation performance was not significantly improved. In addition, when the weight of the Dice loss decreased and the weight of the BCE loss respectively increased, the network segmentation performance was also on a downward trend. These results provide an important reference for further optimizing the training strategy of segmentation networks.

**Table 7:** Segmentation performance results of DEFIF-Net using different weights of the Dice loss (α) and BCE loss (β) in the combined loss function used.

Loss functions’ weights	ISIC2016		ISIC2017		DSB2018	
	IoU (%)	DSC (%)	IoU (%)	DSC (%)	IoU (%)	DSC (%)
α=1; β=0	84.20	90.73	82.61	89.39	80.99	88.68
α=0; β=1	84.05	90.47	82.23	89.15	80.73	88.46
α=0.3; β=0.7	84.36	90.81	82.75	89.66	81.07	88.79
α=0.5; β=0.5	84.72	91.07	82.92	89.83	81.20	88.95
α=0.7; β=0.3 (*used*)	**85.06**	**91.60**	**83.39**	**90.02**	**81.37**	**89.17**

**Fig 10:**
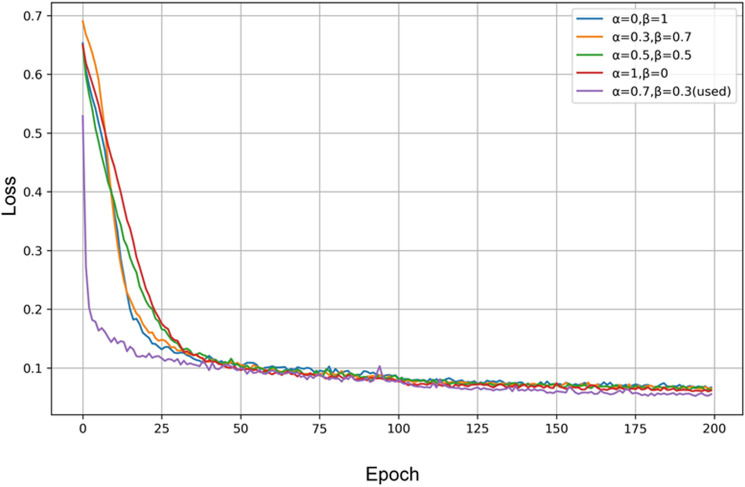
Training curves of the combined loss function using different weights for the Dice loss ( α **) and BCE loss (** β **).**

Fig 10 shows the training curves of the combined loss function using different weights for the Dice loss (α) and BCE loss (β). It can be observed that, initially, the combined loss function rapidly decreases and, after reaching a certain point, it starts to decrease gradually. When α=0.7 and β=0.3 (the weights used in our study), the combined loss is the lowest one, while also exhibiting the fastest convergence.

However, the proposed DEFIF-Net network still has limitations w.r.t. some aspects. First, although DEFIF-Net has achieved a good balance between segmentation performance and efficiency, especially when dealing with medical images with complex backgrounds and subtle structures, it still has to compensate a certain gap in segmentation performance compared with some more complex and heavyweight deep learning networks. These complex networks usually have more parameters and deeper structures, which enable them to capture more subtle image features and achieve top segmentation performance in some challenging tasks. Although DEFIF-Net performs well in most cases, it may still not reach the level of heavyweight networks in extremely complex or detailed image segmentation tasks. Second, in the presented study, DEFIF-Net was mainly evaluated on two types of datasets: skin lesions and cells. Although the utilized datasets cover common scenes in medical images of both types, the performance of the proposed network on other types of medical images, such as CT and MRI images, has not been tested. Therefore, the universality of DEFIF-Net and its application potential in other medical fields still need to be further explored. In the future, we plan to apply DEFIF-Net to a wider range of clinical medical tasks, including but not limited to tumor detection, organ segmentation, blood vessel detection, etc., as to better evaluate its robustness and performance in different medical scenarios.

## 5 Conclusion

This paper has proposed a novel lightweight medical image segmentation network, named DEFIF-Net, with an asymmetric structure. The clever embedding of the newly designed components, presented in the paper, enables DEFIF-Net to achieve top segmentation performance in medical image segmentation tasks. Moreover, these components effectively reduce the network’s parameter count and computational complexity, thus significantly improving the network’s computational speed during the training process. Firstly, to compensate for the absence of spatial features in the network, a novel global dependency fusion (GDF) branch is used as an additional encoder in the proposed network. This branch complements the local features captured by the U-Net original encoder, forming an integrated information structure. Furthermore, this branch effectively fuses distant and nearby feature dependencies by means of a novel feature interaction fusion convolution (FIFConv). Secondly, in the skip connection section of the network, novel channel feature reconstruction modules (CFRMs) are employed to enhance the network’s perception of important channels and facilitate information sharing among different channels. In the bottleneck layer of the network, a novel multi-branch ghost module (MBGM) is employed to ensure the diversity and integrity of the feature information, thus enhancing the network’s robustness and generalization ability. Finally, replacing the U-Net original decoder with the novel residual feature enhancement (RFE) decoder allows the proposed network to focus more on learning and capturing boundary features, thereby improving its adaptability to complex pathological regions. Additionally, employing a BCE−Dice combined loss function during the network training has improved the network’s ability to recognize and segment different pathological categories. Experimental results, obtained on two dermatological public datasets, demonstrated that the proposed network achieves good balance between parameter count, computational complexity, and segmentation performance, displaying state-of-the-art achievement. Additional microscopic cell segmentation experiments demonstrated the strong generalization ability of DEFIF-Net.
